# Effectiveness of Specific Hormonal Protocols on Uterine Involution and Ovarian Cyclicity in Early Postpartum Egyptian Buffaloes

**DOI:** 10.1155/vmi/6584525

**Published:** 2026-04-03

**Authors:** Maha Sabry Salama, Mohey Ahmed Ashour, Abdulrahman Abdulkarim, Mustafa Shukry, Abdel Salam Metwally, Ibrahim Abusaeda, Khaled. A. Khesruf, Arafat Khalphallah, Mohamed Awad Abu El-Hamd

**Affiliations:** ^1^ Department of Diagnostic and Sonography, Animal Reproduction Research Institute (ARRI), Agricultural Research Center (ARC), Giza, Egypt, arc.sci.eg; ^2^ Animal Production Research Institute (APRI), Agricultural Research Center (ARC), Dokki, Giza, Egypt, arc.sci.eg; ^3^ Riwina Animal Production Farm, Agricultural Research Center (ARC), Ministry of Agriculture, Giza, Egypt, agriculture.tn; ^4^ Department of Theriogenology, Faculty of Veterinary Medicine, Omar Almukhtar University, Bayda, Libya; ^5^ Department of Physiology, Faculty of Veterinary Medicine, Kafrelsheikh University, Kafrelsheikh, Egypt, kfs.edu.eg; ^6^ Department of Biomedical Sciences, College of Veterinary Medicine, King Faisal University, Al-Ahsa, Saudi Arabia, kfu.edu.sa; ^7^ Department of Animal Production, Faculty of Agriculture, Kafrelsheikh University, Kafrelsheikh, Egypt, kfs.edu.eg; ^8^ Department of Physiology, Faculty of Veterinary Medicine, Sadat City University, Sadat City, Egypt, usc.edu.eg; ^9^ Department of Animal Diseases, Faculty of Veterinary Medicine, Hama University, Hama, Syria; ^10^ Department of Veterinary Clinical Sciences, Faculty of Veterinary Medicine, Jordan University of Science and Technology, Irbid, Jordan, just.edu.jo

**Keywords:** buffaloes, early postpartum, equine chorionic gonadotropin, gonadotropin-releasing hormone, ovarian cyclicity, reproductive performance, uterine involution

## Abstract

Rapid uterine involution and early resumption of ovarian cyclicity are essential for improving reproductive efficiency in postpartum buffaloes. This study evaluated the effects of different hormonal protocols administered during early postpartum on uterine involution, ovarian activity, and reproductive performance in multiparous Egyptian buffaloes. Particular attention was given to the timing of equine chorionic gonadotropin (eCG) administration, as its application around Day 14 postpartum has been relatively less explored in buffaloes despite coinciding with early uterine recovery and emerging follicular activity. Ninety buffaloes were allocated into five groups receiving gonadotropin‐releasing hormone (GnRH), eCG, or their combinations with prostaglandin F2*α*, in addition to a control group. Reproductive performance, ovarian activity assessed by ultrasonography, and serum progesterone concentrations were monitored during the early postpartum period.

Buffaloes treated with eCG showed shorter uterine involution periods, reduced days open, and higher pregnancy rates compared with untreated controls. Hormonal treatments, particularly eCG‐based protocols, enhanced ovarian cyclicity, as reflected by improved follicular development, corpus luteum formation, and increased progesterone concentrations.

In conclusion, the most favorable reproductive outcomes were achieved when eCG was administered alone or in combination with GnRH and prostaglandin F2*α* during the early postpartum period.

## 1. Introduction

Understanding the pattern of postpartum uterine involution is essential for improving reproductive efficiency and identifying reproductive disorders that negatively impact fertility in buffaloes and dairy cattle [1–3]. Delayed uterine involution prolongs the intercalving interval and significantly reduces fertility in buffaloes [4]. Previous studies have reported that uterine involution in buffaloes is typically completed between 25 and 36 days postpartum, and delays in this process are closely associated with postpartum anestrus and reduced reproductive performance [5]. Consequently, accelerating uterine involution and promoting early resumption of ovarian activity are critical objectives in postpartum reproductive management of buffaloes [6].

Hormonal stimulation plays a pivotal role in initiating ovarian activity during the early postpartum period in buffaloes [7, 8]. The resumption of ovarian cyclicity depends on follicular development and subsequent corpus luteum formation [9]. Equine chorionic gonadotropin (eCG) has been shown to promote follicular growth and ovulation in cattle, thereby improving fertility outcomes [10]. Administration of eCG during the early postpartum period, including as early as Day 6 postpartum, has been reported to reduce the interval from calving to conception [11]. Importantly, eCG administration around Day 14 postpartum has been associated with increased estradiol concentrations and enhanced follicular development, suggesting this time point as a biologically relevant window for intervention [12].

Gonadotropin‐releasing hormone (GnRH) induces the release of luteinizing hormone and follicle‐stimulating hormone, resulting in stimulation of follicular growth, ovulation, and restoration of ovarian cyclicity [13]. Administration of GnRH during the early postpartum period has been shown to improve uterine involution and induce ovarian activity in dairy cows [14]. In addition, prostaglandin F2*α* (PGF2*α*) plays a central role in luteal regression and has been reported to enhance uterine involution, resume ovarian cyclicity, and improve conception rates when administered postpartum [15, 16].

Although GnRH, eCG, and PGF2*α* have each been investigated individually during the postpartum period, their targeted application at specific early postpartum time points remains incompletely explored in buffaloes. In particular, Day 14 postpartum coincides with early uterine recovery and the emergence of follicular activity, making it a physiologically meaningful stage for endocrine manipulation [11, 12]. However, limited information is available regarding the comparative effects of administering eCG alone or in combination with GnRH and PGF2*α* at this time point in buffaloes.

Accordingly, the present study aimed to evaluate the effects of administering GnRH and eCG, either alone or in combination with PGF2*α*, on Day 14 postpartum on uterine involution, ovarian cyclicity, and reproductive performance in Egyptian buffaloes. We hypothesized that hormonal intervention at this early postpartum stage would accelerate uterine involution, enhance ovarian activity, and improve fertility outcomes.

## 2. Materials and Methods

### 2.1. Ethics Approval and Consent to Participate

All animal procedures were approved by the Institutional Animal Care and Use Committee (IACUC) of the Faculty of Veterinary Medicine, Kafrelsheikh University, Egypt (Approval No. IACUC #135/2022) and were conducted in accordance with Egyptian animal welfare regulations and the World Organisation for Animal Health (OIE) standards. The study was designed and reported in compliance with the ARRIVE 2.0 guidelines. All procedures were performed by qualified personnel, animals were monitored regularly, and efforts were made to minimize stress and discomfort.

### 2.2. Animal Management

This study was conducted at the Riwina Animal Production Farm, Agricultural Research Center in Kafrelsheikh, Egypt, from October 2021 to February 2023. Ninety multiparous buffaloes were included in the study, each weighing between 460 and 530 kg, aged 6–8 years, having 2 to 4 parities, and having good body condition scores (BCS; 2.75–3.25). The BCS scale ranged from 1 to 5, with 1 corresponding to emaciated animals and 5 to obese animals [17].

The buffaloes had normal births, with the placentas dropping within 12 h of delivery. They had odorless (noninfected) lochia, were in good physical condition, had a normal uterine appearance, and had been free of common reproductive diseases for at least 2 weeks after giving birth. All animals were housed in open yards, provided with concentrate feed mixtures and roughages following NRC recommendations [18], allowed to graze on Berseem (*Trifolium alexandrinum*) and rice straw during the green season, and had unlimited access to fresh water.

### 2.3. Animals Grouping and Treatment

**FIGURE 1:**
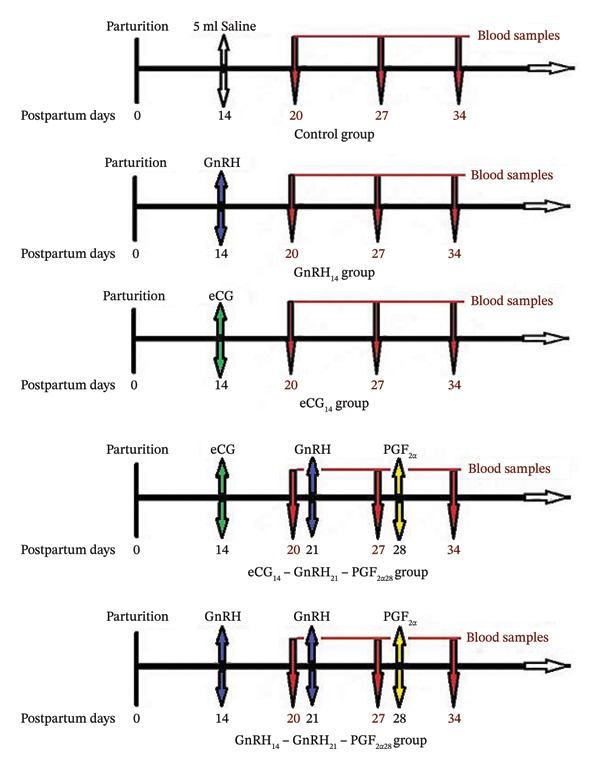
An illustration of the various hormonal therapies used on buffaloes during the early postpartum period. Five groups of 18 buffaloes each were randomly selected. The control group received 5 mL of saline injection on Day 14 postpartum. The GnRH_14_ group received an i.m. Injection of 20 μg of buserelin (5 mL, Receptal) on Day 14 postpartum. The eCG_14_ group received an i.m. Injection of 1000 IU eCG (Gonaser) on Day 14 postpartum. The eCG_14_‐GnRH_21_‐PGF_2α28_ group received an i.m. injection of 1000 IU eCG on Day 14, 5 mL Receptal on Day 21, followed by 750 μg cloprostenol (3 mL Estrumate) on Day 28 postpartum. The GnRH_14_‐GnRH_21_‐PGF_2α28_ group received an i.m. injection of 5 mL Receptal on Days 14 and 21 and 3 mL Estrumate on Day 28 postpartum.

Eligible buffaloes were randomly assigned to the experimental groups using a simple randomization method based on random number generation to ensure unbiased group allocation. All buffaloes (*n* = 90) were divided into five groups of 18 each and represented by flowing lines (Figure 1). The sample size (*n* = 18 per group) was selected based on previous studies investigating postpartum hormonal interventions in buffaloes and dairy cattle that reported significant reproductive and ovarian responses using comparable group sizes [5, 9, 19]. In addition, the number of animals was determined in accordance with ethical principles for animal use and the availability of eligible animals at the experimental facility.

The control group received an intramuscular injection of 5 mL sterile saline (0.9%) on Day 14 postpartum. The GnRH14 group received an intramuscular injection of GnRH (buserelin acetate, 20 μg; Receptal, Intervet International, The Netherlands) on Day 14 postpartum. The eCG14 group received an intramuscular injection of eCG (1000 IU; Gonaser, Laboratorios Hipra, Girona, Spain) on Day 14 postpartum.

The eCG14–GnRH21–PGF2*α*28 (eCG14‐G21‐P28) group received intramuscular injections of eCG (1000 IU) on Day 14 postpartum, GnRH (buserelin acetate, 20 μg) on Day 21 postpartum, and prostaglandin F2*α* (PGF2*α*; cloprostenol sodium, 750 μg; Estrumate, Coopers Animal Health Ltd., Berkhamsted, England) on Day 28 postpartum. The GnRH14–GnRH21–PGF2*α*28 (G14‐G21‐P28) group received intramuscular injections of GnRH (buserelin acetate, 20 μg) on Days 14 and 21 postpartum, followed by PGF2*α* (cloprostenol sodium, 750 μg) on Day 28 postpartum. No animals were euthanized as part of this study; all procedures were nonterminal, and animals remained under routine farm management after the completion of the experiment.

### 2.4. Reproductive Effectiveness

The following reproductive variables were examined: days open (the number of days from calving to conception); days to the first service (the number of days from calving to the first recorded service) [18]; services per conception (the number of matings that are necessary for the buffalo to become pregnant); pregnancy rate (number of pregnant buffaloes/number of naturally mated buffaloes) [20]. The estrous buffaloes that were detected were mated by bulls in December 2021 and January 2022. This occurred at moderate temperatures ranging from 15°C to 25°C during the rainy season. The calving interval (the interval between subsequent calvings) was also monitored [21]. Rectal palpation and ultrasound were used to check all animals for pregnancy 45–50 days after natural breeding, followed by a confirmation at 150 days to avoid early embryonic losses.

### 2.5. Uterine and Ovarian Ultrasonography

Transrectal ultrasonography (SonoScape Co., Ltd., China) was performed with a multifrequency linear transducer (3.0–8.0 MHz) to monitor uterine involution weekly from the second to the seventh postpartum week. The width across the front third of both uterine horns, near the uterine body, was measured dorsoventrally in all animals as part of the study conducted by Kandiel et al. [5]. To monitor uterine shrinkage after birth, researchers measured the diameters of the uterine horns from different angles (ventral, cranial, and dorsal). They also assessed the space between two lines representing the lining of the uterus. This method was developed based on the work of Kähn [22]. Uterine involution was considered complete when both horns could be fully retracted, showed normal echogenicity, and were entirely inside the pelvic cavity [23]. One operator performed all the examinations. The duration for full uterine recovery was determined by calculating the difference between the date of calving and the date of the examination that consistently showed full recovery.

On days 13, 20, 27, and 34 postpartum, both ovaries of each buffalo were observed using transrectal scanning. Each ovary underwent a complete pole‐to‐pole scan, with pictures taken and frozen of various ovarian portions [24]. The follicles within the ovaries were estimated and divided into three categories: small (≤ 5 mm), medium (6–9 mm), and large (≥ 10 mm). The number of corpus lutea within the ovaries was also counted.

### 2.6. Progesterone (P_4_) Assay

Serum progesterone concentrations were evaluated on Days 20, 27, and 34 postpartum. Blood samples were collected into evacuated tubes without an anticoagulant. Immediately after collection, the blood was centrifuged at 1500 × g for 15 min, and the serum was stored at −80°C until progesterone analysis. Progesterone concentrations (ng/mL) were measured using a Coat‐A‐Count solid‐phase radioimmunoassay kit (Coat‐A‐Coat Progesterone, Diagnostic Products Corporation, Los Angeles, CA, USA). The intra‐ and interassay coefficients of variation were 5.1% and 4.2%, respectively, for progesterone. The average sensitivity was 0.09 ng/mL.

### 2.7. Statistical Analysis

The data collected on days open, days to the first service, services per conception, calving interval, and uterine involution days were statistically analyzed using one‐way analysis of variance (ANOVA). Prior to ANOVA, data were tested for normality using the Shapiro–Wilk test and for homogeneity of variances using Levene’s test to confirm that the assumptions of ANOVA were met.

Statistical analyses of serum progesterone concentrations across groups and sampling days were performed using a repeated‐measures ANOVA. Duncan’s multiple‐range test was used for post hoc comparisons of means when significant differences were detected. Pregnancy rate and ovarian follicular and corpus luteum data were expressed as frequencies (percentage of animals exhibiting each structure) and were therefore analyzed using the chi‐square (χ^2^) test. All differences were considered statistically significant at *p* < 0.05.

## 3. Results

### 3.1. Effects of Different Hormone Protocols on Reproductive Performance

Reproductive parameters differed among treatment groups and the control group. Hormonally treated groups exhibited shorter uterine involution periods, fewer days open, earlier first service, and shorter calving intervals compared with the control group. Pregnancy rate varied among groups, with higher proportions of pregnant animals observed in treatment groups. Differences in services per conception were also recorded among groups (Table 1).

**TABLE 1:** Reproductive performance of hormonally treated early postpartum control and experimental buffaloes.

Parameters	Control	GnRH_14_	eCG_14_	eCG_14_‐G_21_‐P_28_	G_14_‐G_21_‐P_28_
Days open	101.71 ± 3.38^a^	81.57 ± 2.14^b^	49.57 ± 1.25^d^	64.14 ± 2.09^c^	68.86 ± 1.75^c^
Days to the first service	95.29 ± 1.49^a^	75.57 ± 1.27^b^	44.43 ± 1.25^d^	59.86 ± 1.01^c^	62.57 ± 1.45^c^
Services per conception	1.56 ± 0.18^a^	1.44 ± 0.18^a^	1.22 ± 0.15^a^	1.33 ± 0.17^a^	1.44 ± 0.18^a^
Pregnancy rate (%)	66.67^c^ (12/18)	72.22^bc^ (13/18)	88.89^a^ (16/18)	83.33^ab^ (15/18)	77.78^abc^ (14/18)
Calving interval	414.29 ± 3.58^a^	396.71 ± 2.24^b^	363.28 ± 1.80^d^	379.29 ± 1.96^c^	383.29 ± 2.02^c^
Uterine involution days	36.57 ± 0.72^a^	33.71 ± 0.87^b^	26.86 ± 0.51^d^	28.71 ± 1.11^cd^	30.29 ± 0.68^c^

*Note:* The number of buffaloes in each group is 18 (*n* = 18), means bearing different superscripts within the same row differed at *p* < 0.05, GnRH_14_ = G_14_ = gonadotropin‐releasing hormone injection on the 14^th^ day; eCG_14_ = equine chorionic gonadotropin injection on the 14^th^ day; G_21_ = gonadotropin‐releasing hormone injection on the 21^st^ day; P_28_ = prostaglandin F_2α_ injection on the 28^th^ day.

### 3.2. Ovarian Structures of Control and Treated Buffaloes During Early Postpartum

Ovarian follicular dynamics differed among groups during the early postpartum period (Tables 2 and 3). On Day 13 postpartum, all animals in all groups exhibited only small follicles (18/18, 100%), with no detection of large follicles or corpus luteum structures.

**TABLE 2:** Small and medium ovarian follicles in the ovaries of control and experimental buffaloes during the early postpartum period.

Parameters	Days	Control	GnRH_14_	eCG_14_	eCG_14_‐G_21_‐P_28_	G_14_‐G_21_‐P_28_
Small follicles	13	100% (18/18)^aA^	100% (18/18)^aA^	100% (18/18)^aA^	100% (18/18)^aA^	100% (18/18)^aA^
	20	100% (18/18)^aA^	50% (9/18)^bB^	44.4% (8/18)^bB^	50% (9/18)^bB^	55.6% (10/18)^bB^
	27	77.8% (14/18)^aB^	44.4% (8/18)^bB^	27.8% (5/18)^cC^	33.3% (6/18)^bcC^	33.3% (6/18)^bcC^
	34	72.2% (13/18)^aB^	44.4% (8/18)^bcB^	33.3% (6/18)^cBC^	44.4% (8/18)^bcBC^	50% (9/18)^bB^

Medium follicles	13	27.8% (5/18)^aB^	27.8% (5/18)^aB^	22.2% (4/18)^aB^	33.3% (6/18)^aB^	22.2% (4/18)^aB^
	20	22.2% (4/18)^cB^	61.1% (11/18)^bA^	77.8% (14/18)^aA^	66.7% (12/18)^abA^	66.7% (12/18)^abA^
	27	44.4% (8/18)^cA^	50% (9/18)^bcA^	66.7% (12/18)^aA^	55.6% (10/18)^abA^	61.1% (11/18)^abA^
	34	55.6% (10/18)^aA^	55.6% (10/18)^aA^	66.7% (12/18)^aA^	66.7% (12/18)^aA^	61.1% (11/18)^aA^

*Note:* The number of buffaloes in each group is 18 (*n* = 18). Values with different superscript letters within the same row (a–d) or within the same column (A–C) differ significantly (*p* < 0.05), GnRH_14_ = G_14_ = gonadotropin‐releasing hormone injection on the 14^th^ day; eCG_14_ = equine chorionic gonadotropin injection on the 14^th^ day; G_21_ = gonadotropin‐releasing hormone injection on the 21^st^ day; P_28_ = prostaglandin F_2α_ injection on the 28^th^ day.

**TABLE 3:** Large follicles and corpus luteum in the ovaries of control and experimental buffaloes during the postpartum period.

Parameters	Days	Control	GnRH_14_	eCG_14_	eCG_14_‐G_21_‐P_28_	G_14_‐G_21_‐P_28_
Large follicles	13	0% (0/18)^aC^	0% (0/18)^aC^	0% (0/18)^aC^	0% (0/18)^aC^	0% (0/18)^aC^
	20	0% (0/18)^bC^	44.4% (8/18)^aB^	55.6% (10/18)^aB^	44.4% (8/18)^aB^	50% (9/18)^aB^
	27	5.6% (1/18)^cB^	50% (9/18)^bAB^	66.7% (12/18)^aB^	55.6% (10/18)^abB^	55.6% (10/18)^abB^
	34	22.2% (4/18)^dA^	61.1% (11/18)^cA^	88.9% (16/18)^aA^	72.2% (13/18)^bcA^	72.2% (13/18)^bcA^

Corpus luteum	13	0% (0/18)^aB^	0% (0/18)^aD^	0% (0/18)^aC^	0% (0/18)^aC^	0% (0/18)^aC^
	20	0% (0/18)^dB^	16.7% (3/18)^bcC^	33.3% (6/18)^aB^	27.8% (5/18)^abB^	16.7% (3/18)^bcB^
	27	5.6% (1/18)^dA^	33.3% (6/18)^bcB^	55.6% (10/18)^aA^	44.4% (8/18)^abA^	44.4% (8/18)^abA^
	34	5.6% (1/18)^dA^	50% (9/18)^bA^	66.7% (12/18)^aA^	27.8% (5/18)^cB^	16.7% (3/18)^cdB^

*Note:* The number of buffaloes in each group is 18 (*n* = 18), values with different superscript letters within the same row (a–d) or within the same column (A–C) differ significantly (*p* < 0.05), GnRH_14_ = G_14_ = gonadotropin‐releasing hormone injection on the 14^th^ day; eCG_14_ = equine chorionic gonadotropin injection on the 14^th^ day; G_21_ = gonadotropin‐releasing hormone injection on the 21^st^ day; P_28_ = prostaglandin F_2α_ injection on the 28^th^ day.

By Day 20 postpartum, the proportion of animals with small follicles decreased in all hormonally treated groups, whereas all control animals continued to exhibit small follicles (18/18, 100%). Specifically, small follicles were observed in 9/18 (50%) animals in the GnRH14 group, 8/18 (44.4%) in the eCG14 group, 9/18 (50%) in the eCG14‐G21‐P28 group, and 10/18 (55.6%) in the G14‐G21‐P28 group (Table 2). Concurrently, medium and large follicles were detected in treated groups, whereas large follicles were not observed in the control group on this day (Table 3).

On Day 27 postpartum, the proportion of animals with small follicles further declined in treated groups, being recorded in 8/18 (44.4%), 5/18 (27.8%), 6/18 (33.3%), and 6/18 (33.3%) animals in the GnRH14, eCG14, eCG14‐G21‐P28, and G14‐G21‐P28 groups, respectively, compared with 14/18 (77.8%) in the control group (Table 2). Medium and large follicles were observed in all groups, with variation in their distribution among treatments (Tables 2 and 3).

By Day 34 postpartum, large follicles were detected in all groups, with proportions ranging from 4/18 (22.2%) in the control group to 16/18 (88.9%) in the eCG14 group (Table 3). Medium follicles were observed in 10/18–12/18 animals across all groups, while small follicles persisted in varying proportions (Table 2). Differences among groups and sampling days were considered statistically significant when superscripts differed (*p* < 0.05), as indicated in Tables 2 and 3.

Corpus luteum structures were not detected in any group on Day 13 postpartum (Table 3). On Day 20 postpartum, corpus luteum was observed in treated groups, being detected in 3/18 (16.7%) animals in the GnRH14 group, 6/18 (33.3%) in the eCG14 group, 5/18 (27.8%) in the eCG14‐G21‐P28 group, and 3/18 (16.7%) in the G14‐G21‐P28 group, while remaining absent in the control group. On Days 27 and 34 postpartum, corpus luteum presence increased in all groups, with variations in frequency among treatments and sampling days (Table 3).

### 3.3. Serum Progesterone Concentration (P_4_) at 20, 27, and 34 days After the Administration of Different Protocols

Serum progesterone concentrations differed among groups and sampling days (Table 4). On Day 20 postpartum, progesterone concentrations were lower in the control group and higher in hormonally treated groups. By Day 27 postpartum, progesterone concentrations increased in all groups, with treated groups exhibiting higher values than the control group. On Day 34 postpartum, progesterone concentrations remained elevated in the GnRH14 and eCG14 groups, whereas lower concentrations were recorded in groups receiving PGF2*α* (Table 4). Differences among groups and across sampling days were considered statistically significant when superscripts differed (*p* < 0.05), as indicated in Table 4.

**TABLE 4:** Serum progesterone concentrations in control and experimental buffaloes.

Parameters	Days	Control	GnRH_14_	eCG_14_	eCG_14_‐G_21_‐P_28_	G_14_‐G_21_‐P_28_
P_4_ concentrations (ng/mL)	20	0.37 ± 0.02^cC^	0.55 ± 0.04^bC^	0.74 ± 0.03^aC^	0.63 ± 0.05^abC^	0.54 ± 0.03^bC^
	27	0.54 ± 0.04^cB^	0.94 ± 0.04^bB^	1.26 ± 0.06^aB^	1.03 ± 0.05^bA^	0.99 ± 0.05^bA^
	34	0.67 ± 0.02^dA^	1.35 ± 0.03^bA^	1.51 ± 0.07^aA^	0.87 ± 0.04^cB^	0.79 ± 0.20^cB^

*Note:* The number of buffaloes in each group is 18 (*n* = 18), means with different superscript letters within the same row (a–d) or within the same column (A–C) are significantly different (*p* < 0.05), P_4_ = progesterone; GnRH_14_ = G_14_ = gonadotropin‐releasing hormone injection on the 14^th^ day; G_21_ = gonadotropin‐releasing hormone injection on the 21^st^ day; P_28_ = prostaglandin F_2α_ injection on the 28^th^ day; eCG_14_ = equine chorionic gonadotropin injection on the 14^th^ day.

## 4. Discussion

The present study demonstrates that initiating hormonal intervention on Day 14 postpartum effectively accelerates uterine involution and promotes early resumption of ovarian cyclicity in Egyptian buffaloes. This point appears to represent a physiologically responsive window, coinciding with early uterine tissue repair and the emergence of initial postpartum follicular waves. At this stage, ovarian follicles regain sensitivity to gonadotropins, allowing endocrine treatments to exert maximal effects on follicular growth and subsequent reproductive recovery [5, 9, 11].

The observed acceleration of uterine involution in hormonally treated buffaloes is consistent with previous reports indicating that early postpartum endocrine manipulation enhances uterine clearance and functional recovery [25, 26]. Compared with studies reporting slower involution or more modest reproductive responses [17, 27], the improved outcomes observed in the present study may be explained by differences in buffalo breed, parity, nutritional management, and environmental conditions. Animals included in this study exhibited optimal BCS and were managed under controlled feeding and housing systems, factors known to enhance endocrine responsiveness during the postpartum period [3, 5]. In addition, the timing and dosage of hormonal administration may have contributed to the greater magnitude of response observed, as early intervention may prevent the establishment of prolonged postpartum anestrus [11, 12].

Enhancement of ovarian activity following GnRH and eCG administration was evident through earlier follicular development and corpus luteum formation, supporting previous findings in buffaloes and dairy cattle [9, 19, 28]. The superior response observed in eCG‐treated animals may reflect the dual FSH‐ and LH‐like activity of eCG, which promotes follicular growth and steroidogenesis more effectively than GnRH alone [12, 29], compared with studies that administrate hormonal treatments later in the postpartum period [14, 26]. Early stimulation at Day 14 postpartum may amplify ovarian steroid output before hypothalamic–pituitary suppression becomes pronounced [27].

Mechanistically, early restoration of ovarian cyclicity enhances estradiol secretion, which plays a key role in stimulating uterine contractility and facilitating uterine involution [12]. This endocrine response supports effective uterine clearance and functional recovery, thereby creating a favorable uterine environment for subsequent conception. In the present study, mechanistic interpretation was intentionally focused on these core endocrine pathways to avoid unnecessary elaboration while maintaining biological relevance.

Improved reproductive performance in treated buffaloes, including reduced days open, earlier first service, and shorter calving intervals, likely reflects the combined effects of accelerated uterine recovery and early ovarian reactivation rather than isolated hormonal action [30]. Early establishment of ovarian cyclicity minimizes the duration of postpartum anestrus, enhances estrus expression, and improves conception efficiency. From a management perspective, these outcomes are particularly important in buffalo production systems, where prolonged postpartum infertility represents a major economic constraint [30, 31].

Differences in pregnancy rate and progesterone profiles between treatment groups further support the importance of protocol design. Higher progesterone concentrations observed in eCG‐treated animals may be attributed to enhanced corpus luteum development and luteal function, as previously reported [32, 33]. In contrast, reduced progesterone concentrations following PGF2*α* administration reflect its expected luteolytic action, facilitating estrous synchronization and reproductive turnover [16, 19].

Overall, the present findings highlight the importance of early postpartum hormonal intervention tailored to the physiological status of buffaloes. Administering eCG, alone or in combination with GnRH and PGF2*α*, at Day 14 postpartum appears to optimize uterine recovery, ovarian activity, and reproductive efficiency more effectively than later interventions. Variations among studies underscore the influence of breed, management, and protocol timing on reproductive outcomes, emphasizing the need for context‐specific reproductive strategies in buffalo herds.

## 5. Conclusion

Early postpartum hormonal intervention at Day 14 postpartum supports coordinated uterine recovery and early resumption of ovarian cyclicity in buffaloes, as demonstrated in the present study. Under the conditions investigated, protocols involving eCG alone or in combination with GnRH and PGF2*α* were associated with improved reproductive efficiency, without indicating definitive superiority over other approaches. As discussed earlier, these effects may contribute to practical reproductive benefits at the herd level, including improved fertility performance and potential shortening of the calving interval under farm conditions. Overall, the findings highlight the value of strategically timed hormonal management during the early postpartum period as a practical tool for improving reproductive outcomes in buffalo production systems.

## Author Contributions

Maha Sabry Salama: conceptualization, methodology, investigation, visualization, formal analysis, writing–original draft, and writing–review and editing. Mohey Ahmed Ashour: conceptualization, methodology, investigation, data curation, visualization, formal analysis. Abdulrahman Abdulkarim: methodology, software, formal analysis, and writing–original draft. Mustafa Shukry: conceptualization, investigation, supervision, and writing–review and editing, validation. Abdel Salam Metwally: conceptualization, methodology, software, formal analysis, and writing–original draft. Ibrahim Abusaeda: conceptualization, methodology, investigation, data curation, validation, and writing–review and editing. Khaled A. Khesruf: methodology, investigation, software, formal analysis, and writing–original draft. Arafat Khalphallah: conceptualization, investigation, visualization, and writing–original draft. Mohamed Awad Abu El‐Hamd: conceptualization, methodology, investigation, supervision, writing–original draft, and writing–review and editing.

## Funding

The study did not receive any external fund.

## Disclosure

All authors have read and approved the final manuscript.

## Conflicts of Interest

The authors declare no conflicts of interest.

## Data Availability

The datasets used and/or analyzed during the current study are available from the corresponding author on reasonable request.
